# 3-(4-Bromo­phenyl­sulfon­yl)-8-methyl-1,3-diaza­spiro­[4.5]decane-2,4-dione

**DOI:** 10.1107/S1600536809027305

**Published:** 2009-07-18

**Authors:** M. Kalim Kashif, M. Khawar Rauf, Michael Bolte, Shahid Hameed

**Affiliations:** a Department of Chemistry, Quaid-i-Azam University, Islamabad 45320, Pakistan; b Institut für Anorganische Chemie, J. W. Goethe-Universität Frankfurt, Max-von-Laue-Strasse 7, 60438 Frankfurt/Main, Germany

## Abstract

The crystal structure of the title compound, C_15_H_17_BrN_2_O_4_S, is stabilized by inter­molecular N—H⋯O hydrogen bonds which link the mol­ecules into centrosymmetric dimers. The dihedral angle subtended by the 4-bromo­phenyl group with the mean plane passing through the hydantoin unit is 83.29 (5)°. The cyclo­hexyl group adopts an ideal chair conformation with the methyl group in an equatorial position.

## Related literature

For background to diabetes and its treatment, see: Tiwari & Rao (2002 ▶); DeFronzo (1999 ▶); Feinglos & Bethel (1998 ▶); Murakami *et al.*, (1997 ▶); Kashif, Ahmad *et al.* (2008 ▶). For related structures, see: Gauthier *et al.* (1997 ▶); Hussain *et al.* (2009*a* ▶,*b* ▶); Kashif, Hussain *et al.* (2008 ▶).
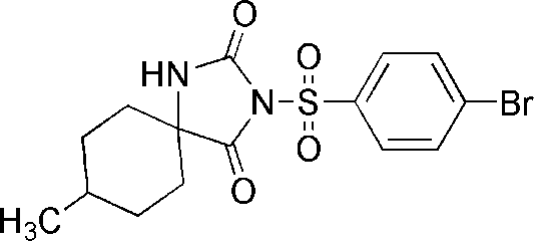


## Experimental

### 

#### Crystal data

C_15_H_17_BrN_2_O_4_S
*M* *_r_* = 401.28
Monoclinic, 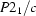

*a* = 6.1835 (5) Å
*b* = 17.5442 (9) Å
*c* = 15.1865 (11) Å
β = 94.222 (6)°
*V* = 1643.0 (2) Å^3^
*Z* = 4
Mo *K*α radiation
μ = 2.65 mm^−1^
*T* = 173 K
0.26 × 0.26 × 0.23 mm

#### Data collection

Stoe IPDS-II two-circle diffractometer
Absorption correction: multi-scan (*MULABS*; Spek, 2009 ▶; Blessing, 1995 ▶) *T*_min_ = 0.546, *T*_max_ = 0.581 (expected range = 0.511–0.544)
25973 measured reflections
3580 independent reflections
3067 reflections with *I* > 2σ(*I*)
*R*_int_ = 0.053

#### Refinement

*R*[*F* ^2^ > 2σ(*F* ^2^)] = 0.030
*wR*(*F* ^2^) = 0.070
*S* = 1.03
3580 reflections
213 parameters
H atoms treated by a mixture of independent and constrained refinement
Δρ_max_ = 0.32 e Å^−3^
Δρ_min_ = −0.46 e Å^−3^

### 

Data collection: *X-AREA* (Stoe & Cie, 2001 ▶); cell refinement: *X-AREA*; data reduction: *X-AREA*; program(s) used to solve structure: *SHELXS97* (Sheldrick, 2008 ▶); program(s) used to refine structure: *SHELXL97* (Sheldrick, 2008 ▶); molecular graphics: *XP* in *SHELXTL-Plus* (Sheldrick, 2008 ▶); software used to prepare material for publication: *SHELXL97*.

## Supplementary Material

Crystal structure: contains datablocks I, global. DOI: 10.1107/S1600536809027305/pv2181sup1.cif

Structure factors: contains datablocks I. DOI: 10.1107/S1600536809027305/pv2181Isup2.hkl

Additional supplementary materials:  crystallographic information; 3D view; checkCIF report

## Figures and Tables

**Table 1:** Hydrogen-bond geometry (Å, °)

*D*—H⋯*A*	*D*—H	H⋯*A*	*D*⋯*A*	*D*—H⋯*A*
N2—H2⋯O4^i^	0.82 (3)	2.06 (3)	2.871 (2)	171 (3)

Symmetry code: (i) 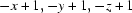.

**Fig. 1.**
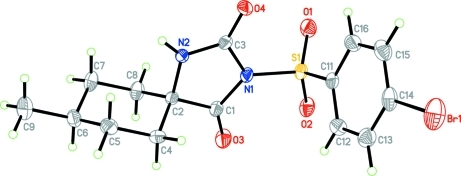
Molecular structure of the title compound with displacement ellipsoids at the 50% probability level showing atom-labelling scheme.

## supplementary crystallographic information

### Comment

Diabetes is one of the major causes of disease related deaths in these modern times and the people in South-East Asia and Western Pacific are being the most at risk (Tiwari & Rao, 2002). To cure the disease sulfonyl ureas are the most frequently used antidiabetic drugs (DeFronzo, 1999; Feinglos & Bethel, 1998). An important complication related to this disease is the cataract formation and imidazolidine-2,4-diones have been found as aldose reductase inhibitors (Murakami *et al.*, 1997). The combination of the two scaffolds, *i.e.* the sulfonyl urea and the imidazolidine-2,4-dione, in one molecule may be a useful combination to cure the disease and associated complications, especially the cataract formation. With this hypothesis in mind, we synthesized a number of *N*-arylsulfonylimidazolidine-2,4-diones and evaluated their antidiabetic activity (Kashif & Ahmad *et al.*, 2008) and their crystal structures (Hussain *et al.*, 2009*a*; Hussain *et al.*, 2009*b*; Kashif & Hussain *et al.*, 2008). In this article, we report the synthesis and crystal structure of the title compound.

The structure of the title compound is presented in Fig. 1. The bond lengths and angles within the hydantoin (2,4-imidazolidenedione) moiety are normal, typical of those observed in cyclohexanespiro-5'-hydantoin (Gauthier *et al.*, 1997; Kashif, Hussain *et al.*, 2008). The hydantoin unit is planar (r.m.s. deviation of 0.007 Å). The cyclohexane ring adopts an ideal chair conformation with the methyl group in an equatorial position. The endocyclic torsion angles range from 55.2 (2) to 56.4 (2)°. The C1—O3 and C3—O4 bond lengths are 1.199 (2) and 1.226 (2) Å, respectively, which are close to the standard value for C═O (1.20 Å). The dihedral angle subtended by the *p*-bromophenyl group with the plane passing through the hydantoin moiety is 83.29 (5)°. Intermolecular N—H···O hydrogen bonds link the molecules to centrosymmetric dimers (Table 1).

### Experimental

Substituted cyclohexanone (0.1 mol) and ammonium carbonate (0.6 mol) were placed in a 100 ml round bottom flask. Potassium cyanide (0.1 mol) was dissolved in aqueous ethanol (60%) and added to the reaction flask. The mixture was heated on an oil bath at 328–333 K until the reaction was complete (monitored by *TLC*). After cooling to room temperature, the reaction mixture was concentrated and acidified using conc. HCl. The resulting precipitates were filtered, dissolved in saturated NaOH_(aq)_ solution and extracted with diethyl ether (2 × 25 ml). The aqueous layer was acidified to precipitate 8-substituted-1,3-diazaspiro[4.5]decane-2,4-dione, which was filtered and recrystallized from ethanol/water. 8-substituted-1,3-diazaspiro[4.5]decane-2,4-dione (4.8 mmol) in CH_2_Cl_2_ (20 ml) was stirred with triethylamine (4.8 mmol) and catalytic amounts of dimethylaminopyridine (DMAP). The 4-bromobenzene sulfonyl chloride (5.8 mmol) in CH_2_Cl_2_ (10 ml) was added drop wise and the reaction mixture stirred at room temperature. After completion of the reaction (*TLC*), the mixture was diluted with 1 *M* HCl (20 ml) and extracted with CH_2_Cl_2_ (3 × 25 ml). The organic layer was dried over anhydrous sodium sulfate and concentrated under reduced pressure. Recrystallization of the residue from ethyl acetate afforded the colourless plate-like crystals, suitable for X-ray analysis.

### Refinement

H atom on the N atom was refined isotropically. Other H atoms were placed in idealized positions and treated as riding atoms with C—H distance in the range 0.95–1.00 Å and *U*_iso_(H) = 1.2*U*_eq_(C) or 1.5*U*_eq_(C_methyl_).

### Crystal data

**Table:**

C_15_H_17_BrN_2_O_4_S	*F*(000) = 816
*M**_r_* = 401.28	*D*_x_ = 1.622 Mg m^−^^3^
Monoclinic, *P*2_1_/*c*	Mo *K*α radiation, λ = 0.71073 Å
Hall symbol: -P 2ybc	Cell parameters from 21668 reflections
*a* = 6.1835 (5) Å	θ = 3.0–27.3°
*b* = 17.5442 (9) Å	µ = 2.65 mm^−^^1^
*c* = 15.1865 (11) Å	*T* = 173 K
β = 94.222 (6)°	Block, colourless
*V* = 1643.0 (2) Å^3^	0.26 × 0.26 × 0.23 mm
*Z* = 4	

### Data collection

**Table:**

Stoe IPDS-II two-circle diffractometer	3580 independent reflections
Radiation source: fine-focus sealed tube	3067 reflections with *I* > 2σ(*I*)
graphite	*R*_int_ = 0.053
ω scans	θ_max_ = 27.1°, θ_min_ = 2.9°
Absorption correction: multi-scan (*MULABS*; Spek, 2009; Blessing, 1995)	*h* = −6→7
*T*_min_ = 0.546, *T*_max_ = 0.581	*k* = −22→22
25973 measured reflections	*l* = −19→19

### Refinement

**Table:**

Refinement on *F*^2^	Secondary atom site location: difference Fourier map
Least-squares matrix: full	Hydrogen site location: inferred from neighbouring sites
*R*[*F*^2^ > 2σ(*F*^2^)] = 0.030	H atoms treated by a mixture of independent and constrained refinement
*wR*(*F*^2^) = 0.070	*w* = 1/[σ^2^(*F*_o_^2^) + (0.0362*P*)^2^ + 0.6401*P*] where *P* = (*F*_o_^2^ + 2*F*_c_^2^)/3
*S* = 1.03	(Δ/σ)_max_ = 0.002
3580 reflections	Δρ_max_ = 0.32 e Å^−^^3^
213 parameters	Δρ_min_ = −0.46 e Å^−^^3^
0 restraints	Extinction correction: *SHELXL97* (Sheldrick, 2008), Fc^*^=kFc[1+0.001xFc^2^λ^3^/sin(2θ)]^-1/4^
Primary atom site location: structure-invariant direct methods	Extinction coefficient: 0.0121 (7)

### Special details

**Table:**

Geometry. All e.s.d.'s (except the e.s.d. in the dihedral angle between two l.s. planes) are estimated using the full covariance matrix. The cell e.s.d.'s are taken into account individually in the estimation of e.s.d.'s in distances, angles and torsion angles; correlations between e.s.d.'s in cell parameters are only used when they are defined by crystal symmetry. An approximate (isotropic) treatment of cell e.s.d.'s is used for estimating e.s.d.'s involving l.s. planes.
Refinement. Refinement of *F*^2^ against ALL reflections. The weighted *R*-factor *wR* and goodness of fit *S* are based on *F*^2^, conventional *R*-factors *R* are based on *F*, with *F* set to zero for negative *F*^2^. The threshold expression of *F*^2^ > σ(*F*^2^) is used only for calculating *R*-factors(gt) *etc*. and is not relevant to the choice of reflections for refinement. *R*-factors based on *F*^2^ are statistically about twice as large as those based on *F*, and *R*- factors based on ALL data will be even larger.

### Fractional atomic coordinates and isotropic or equivalent isotropic displacement parameters (Å^2^)

**Table:**

	*x*	*y*	*z*	*U*_iso_*/*U*_eq_	
S1	0.36497 (7)	0.36912 (2)	0.23469 (3)	0.02034 (11)	
Br1	0.79687 (4)	0.639526 (13)	0.026217 (15)	0.04204 (10)	
N1	0.5052 (3)	0.36880 (8)	0.33540 (10)	0.0194 (3)	
N2	0.6270 (2)	0.41482 (9)	0.46652 (10)	0.0192 (3)	
H2	0.641 (4)	0.4424 (15)	0.5103 (18)	0.039 (7)*	
O1	0.1450 (2)	0.38760 (9)	0.24820 (9)	0.0300 (3)	
O2	0.4194 (2)	0.29902 (8)	0.19481 (9)	0.0279 (3)	
O3	0.7063 (3)	0.25497 (8)	0.33600 (9)	0.0348 (4)	
O4	0.3747 (2)	0.48633 (7)	0.38287 (9)	0.0257 (3)	
C1	0.6576 (3)	0.31357 (10)	0.37006 (12)	0.0210 (4)	
C2	0.7465 (3)	0.34343 (10)	0.45976 (11)	0.0178 (3)	
C3	0.4922 (3)	0.43063 (10)	0.39634 (11)	0.0183 (3)	
C4	0.9913 (3)	0.35923 (11)	0.45864 (12)	0.0243 (4)	
H4A	1.0673	0.3118	0.4436	0.029*	
H4B	1.0163	0.3978	0.4129	0.029*	
C5	1.0824 (3)	0.38812 (12)	0.54880 (13)	0.0274 (4)	
H5A	1.0162	0.4381	0.5608	0.033*	
H5B	1.2408	0.3957	0.5476	0.033*	
C6	1.0387 (3)	0.33271 (12)	0.62327 (13)	0.0292 (4)	
H6	1.1133	0.2835	0.6116	0.035*	
C7	0.7960 (4)	0.31675 (12)	0.62351 (13)	0.0286 (4)	
H7A	0.7203	0.3641	0.6387	0.034*	
H7B	0.7714	0.2783	0.6694	0.034*	
C8	0.6999 (3)	0.28756 (11)	0.53384 (12)	0.0255 (4)	
H8A	0.5413	0.2811	0.5357	0.031*	
H8B	0.7632	0.2372	0.5215	0.031*	
C9	1.1315 (4)	0.36296 (16)	0.71236 (15)	0.0443 (6)	
H9A	1.2873	0.3722	0.7101	0.067*	
H9B	1.1080	0.3254	0.7584	0.067*	
H9C	1.0588	0.4108	0.7257	0.067*	
C11	0.4836 (3)	0.44498 (10)	0.17971 (11)	0.0196 (3)	
C12	0.6799 (3)	0.43179 (11)	0.14276 (13)	0.0256 (4)	
H12	0.7492	0.3836	0.1489	0.031*	
C13	0.7725 (3)	0.49032 (12)	0.09680 (13)	0.0286 (4)	
H13	0.9069	0.4829	0.0714	0.034*	
C14	0.6662 (3)	0.55965 (11)	0.08849 (12)	0.0270 (4)	
C15	0.4704 (4)	0.57335 (11)	0.12458 (13)	0.0294 (4)	
H15	0.4007	0.6215	0.1176	0.035*	
C16	0.3782 (3)	0.51486 (11)	0.17143 (13)	0.0258 (4)	
H16	0.2448	0.5227	0.1974	0.031*	

### Atomic displacement parameters (Å^2^)

**Table:**

	*U*^11^	*U*^22^	*U*^33^	*U*^12^	*U*^13^	*U*^23^
S1	0.0226 (2)	0.0223 (2)	0.0156 (2)	−0.00345 (16)	−0.00226 (16)	−0.00205 (15)
Br1	0.05839 (18)	0.03393 (13)	0.03336 (13)	−0.01601 (10)	0.00050 (10)	0.00848 (9)
N1	0.0248 (8)	0.0191 (7)	0.0140 (6)	0.0017 (6)	−0.0009 (6)	−0.0035 (5)
N2	0.0213 (8)	0.0199 (7)	0.0159 (7)	0.0055 (6)	−0.0018 (6)	−0.0047 (6)
O1	0.0209 (7)	0.0426 (8)	0.0260 (7)	−0.0039 (6)	−0.0020 (6)	0.0011 (6)
O2	0.0392 (8)	0.0227 (7)	0.0208 (7)	−0.0040 (6)	−0.0031 (6)	−0.0047 (5)
O3	0.0560 (10)	0.0231 (7)	0.0242 (7)	0.0139 (7)	−0.0041 (7)	−0.0080 (5)
O4	0.0295 (7)	0.0247 (7)	0.0221 (6)	0.0116 (5)	−0.0042 (5)	−0.0050 (5)
C1	0.0276 (10)	0.0188 (8)	0.0167 (8)	0.0012 (7)	0.0018 (7)	0.0005 (6)
C2	0.0212 (9)	0.0163 (8)	0.0157 (8)	0.0031 (6)	0.0014 (6)	0.0000 (6)
C3	0.0200 (8)	0.0195 (8)	0.0155 (8)	0.0004 (6)	0.0018 (6)	−0.0027 (6)
C4	0.0196 (9)	0.0299 (9)	0.0240 (9)	0.0051 (7)	0.0057 (7)	0.0005 (7)
C5	0.0170 (9)	0.0357 (10)	0.0294 (10)	−0.0013 (8)	0.0001 (7)	−0.0036 (8)
C6	0.0325 (11)	0.0314 (10)	0.0225 (9)	0.0098 (8)	−0.0065 (8)	−0.0031 (8)
C7	0.0385 (12)	0.0306 (10)	0.0166 (8)	−0.0042 (8)	0.0010 (8)	0.0042 (7)
C8	0.0331 (11)	0.0228 (9)	0.0204 (9)	−0.0061 (8)	−0.0001 (8)	0.0039 (7)
C9	0.0427 (13)	0.0586 (16)	0.0296 (11)	0.0032 (11)	−0.0113 (10)	−0.0099 (11)
C11	0.0221 (9)	0.0209 (8)	0.0151 (8)	−0.0008 (7)	−0.0022 (7)	−0.0008 (6)
C12	0.0254 (10)	0.0247 (9)	0.0266 (9)	0.0031 (7)	0.0009 (8)	0.0013 (7)
C13	0.0264 (10)	0.0336 (11)	0.0260 (10)	−0.0031 (8)	0.0031 (8)	0.0020 (8)
C14	0.0353 (11)	0.0252 (9)	0.0194 (8)	−0.0086 (8)	−0.0061 (8)	0.0018 (7)
C15	0.0363 (11)	0.0221 (9)	0.0288 (10)	0.0029 (8)	−0.0049 (8)	0.0010 (8)
C16	0.0269 (10)	0.0258 (10)	0.0242 (9)	0.0042 (8)	−0.0021 (8)	−0.0025 (7)

### Geometric parameters (Å, °)

**Table:**

S1—O2	1.4222 (14)	C6—C9	1.525 (3)
S1—O1	1.4279 (15)	C6—C7	1.527 (3)
S1—N1	1.7018 (16)	C6—H6	1.0000
S1—C11	1.7597 (18)	C7—C8	1.533 (3)
Br1—C14	1.9035 (19)	C7—H7A	0.9900
N1—C1	1.425 (2)	C7—H7B	0.9900
N1—C3	1.432 (2)	C8—H8A	0.9900
N2—C3	1.333 (2)	C8—H8B	0.9900
N2—C2	1.461 (2)	C9—H9A	0.9800
N2—H2	0.82 (3)	C9—H9B	0.9800
O3—C1	1.199 (2)	C9—H9C	0.9800
O4—C3	1.226 (2)	C11—C16	1.390 (3)
C1—C2	1.524 (2)	C11—C12	1.394 (3)
C2—C8	1.535 (2)	C12—C13	1.389 (3)
C2—C4	1.540 (3)	C12—H12	0.9500
C4—C5	1.528 (3)	C13—C14	1.384 (3)
C4—H4A	0.9900	C13—H13	0.9500
C4—H4B	0.9900	C14—C15	1.386 (3)
C5—C6	1.530 (3)	C15—C16	1.394 (3)
C5—H5A	0.9900	C15—H15	0.9500
C5—H5B	0.9900	C16—H16	0.9500
			
O2—S1—O1	121.02 (9)	C9—C6—H6	108.0
O2—S1—N1	105.01 (8)	C7—C6—H6	108.0
O1—S1—N1	107.43 (8)	C5—C6—H6	108.0
O2—S1—C11	109.39 (8)	C6—C7—C8	112.09 (16)
O1—S1—C11	109.34 (9)	C6—C7—H7A	109.2
N1—S1—C11	103.08 (8)	C8—C7—H7A	109.2
C1—N1—C3	110.08 (14)	C6—C7—H7B	109.2
C1—N1—S1	127.85 (12)	C8—C7—H7B	109.2
C3—N1—S1	121.95 (12)	H7A—C7—H7B	107.9
C3—N2—C2	114.31 (14)	C7—C8—C2	110.87 (15)
C3—N2—H2	123.2 (19)	C7—C8—H8A	109.5
C2—N2—H2	122.4 (19)	C2—C8—H8A	109.5
O3—C1—N1	127.13 (18)	C7—C8—H8B	109.5
O3—C1—C2	126.52 (18)	C2—C8—H8B	109.5
N1—C1—C2	106.35 (14)	H8A—C8—H8B	108.1
N2—C2—C1	101.93 (14)	C6—C9—H9A	109.5
N2—C2—C8	111.88 (14)	C6—C9—H9B	109.5
C1—C2—C8	111.15 (15)	H9A—C9—H9B	109.5
N2—C2—C4	110.41 (15)	C6—C9—H9C	109.5
C1—C2—C4	110.00 (14)	H9A—C9—H9C	109.5
C8—C2—C4	111.13 (15)	H9B—C9—H9C	109.5
O4—C3—N2	128.86 (16)	C16—C11—C12	121.77 (17)
O4—C3—N1	123.83 (16)	C16—C11—S1	120.07 (14)
N2—C3—N1	107.31 (15)	C12—C11—S1	118.12 (14)
C5—C4—C2	110.35 (14)	C13—C12—C11	118.91 (18)
C5—C4—H4A	109.6	C13—C12—H12	120.5
C2—C4—H4A	109.6	C11—C12—H12	120.5
C5—C4—H4B	109.6	C14—C13—C12	119.00 (19)
C2—C4—H4B	109.6	C14—C13—H13	120.5
H4A—C4—H4B	108.1	C12—C13—H13	120.5
C4—C5—C6	112.21 (17)	C13—C14—C15	122.63 (18)
C4—C5—H5A	109.2	C13—C14—Br1	118.44 (16)
C6—C5—H5A	109.2	C15—C14—Br1	118.93 (15)
C4—C5—H5B	109.2	C14—C15—C16	118.43 (18)
C6—C5—H5B	109.2	C14—C15—H15	120.8
H5A—C5—H5B	107.9	C16—C15—H15	120.8
C9—C6—C7	111.54 (18)	C11—C16—C15	119.26 (18)
C9—C6—C5	111.05 (19)	C11—C16—H16	120.4
C7—C6—C5	110.20 (16)	C15—C16—H16	120.4
			
O2—S1—N1—C1	4.51 (18)	C8—C2—C4—C5	−55.7 (2)
O1—S1—N1—C1	134.56 (16)	C2—C4—C5—C6	56.4 (2)
C11—S1—N1—C1	−110.02 (16)	C4—C5—C6—C9	179.97 (18)
O2—S1—N1—C3	−179.83 (14)	C4—C5—C6—C7	−55.9 (2)
O1—S1—N1—C3	−49.78 (16)	C9—C6—C7—C8	179.05 (18)
C11—S1—N1—C3	65.64 (15)	C5—C6—C7—C8	55.2 (2)
C3—N1—C1—O3	179.69 (19)	C6—C7—C8—C2	−55.5 (2)
S1—N1—C1—O3	−4.2 (3)	N2—C2—C8—C7	−68.6 (2)
C3—N1—C1—C2	0.03 (19)	C1—C2—C8—C7	178.19 (16)
S1—N1—C1—C2	176.10 (12)	C4—C2—C8—C7	55.3 (2)
C3—N2—C2—C1	−1.37 (19)	O2—S1—C11—C16	146.67 (15)
C3—N2—C2—C8	−120.20 (17)	O1—S1—C11—C16	12.04 (18)
C3—N2—C2—C4	115.47 (17)	N1—S1—C11—C16	−102.01 (16)
O3—C1—C2—N2	−178.93 (19)	O2—S1—C11—C12	−31.16 (17)
N1—C1—C2—N2	0.74 (18)	O1—S1—C11—C12	−165.79 (14)
O3—C1—C2—C8	−59.6 (3)	N1—S1—C11—C12	80.16 (15)
N1—C1—C2—C8	120.08 (16)	C16—C11—C12—C13	0.3 (3)
O3—C1—C2—C4	63.9 (2)	S1—C11—C12—C13	178.06 (15)
N1—C1—C2—C4	−116.41 (16)	C11—C12—C13—C14	−0.5 (3)
C2—N2—C3—O4	−178.87 (18)	C12—C13—C14—C15	0.2 (3)
C2—N2—C3—N1	1.4 (2)	C12—C13—C14—Br1	179.43 (15)
C1—N1—C3—O4	179.42 (17)	C13—C14—C15—C16	0.3 (3)
S1—N1—C3—O4	3.1 (2)	Br1—C14—C15—C16	−178.85 (15)
C1—N1—C3—N2	−0.9 (2)	C12—C11—C16—C15	0.3 (3)
S1—N1—C3—N2	−177.22 (12)	S1—C11—C16—C15	−177.44 (15)
N2—C2—C4—C5	69.10 (19)	C14—C15—C16—C11	−0.6 (3)
C1—C2—C4—C5	−179.18 (15)		

### Hydrogen-bond geometry (Å, °)

**Table:**

*D*—H···*A*	*D*—H	H···*A*	*D*···*A*	*D*—H···*A*
N2—H2···O4^i^	0.82 (3)	2.06 (3)	2.871 (2)	171 (3)

Symmetry codes: (i) −*x*+1, −*y*+1, −*z*+1.
